# S6 kinase 1 at the central node of cell size and ageing

**DOI:** 10.3389/fcell.2022.949196

**Published:** 2022-08-12

**Authors:** Stefano Fumagalli, Mario Pende

**Affiliations:** Université Paris Cité, INSERM UMR-S1151, CNRS UMR-S8253, Institut Necker Enfants Malades, Paris, France

**Keywords:** mTOR, signal transduction, nutrition, growth, ageing, senescence

## Abstract

Genetic evidence in living organisms from yeast to plants and animals, including humans, unquestionably identifies the Target Of Rapamycin kinase (TOR or mTOR for mammalian/mechanistic) signal transduction pathway as a master regulator of growth through the control of cell size and cell number. Among the mTOR targets, the activation of p70 S6 kinase 1 (S6K1) is exquisitely sensitive to nutrient availability and rapamycin inhibition. Of note, *in vivo* analysis of mutant flies and mice reveals that S6K1 predominantly regulates cell size versus cell proliferation. Here we review the putative mechanisms of S6K1 action on cell size by considering the main functional categories of S6K1 targets: substrates involved in nucleic acid and protein synthesis, fat mass accumulation, retrograde control of insulin action, senescence program and cytoskeleton organization. We discuss how S6K1 may be involved in the observed interconnection between cell size, regenerative and ageing responses.

## Genetic evidence of growth regulators and signal transduction mechanisms

At the turn of the second and third millennium, genetic screens for tissue overgrowth in *Drosophila* flies identify loss-of-function (LOF) mutants falling in two broad categories: hyperplastic with increased cell size, such as Gigas-Tuberous Sclerosis Complex 2 (*TSC2*), Tuberous Sclerosis Complex 1 (*TSC1*) and Phosphatase And Tensin Homolog (*PTEN*) (Huang et al., 1999; Ito and Rubin, 1999; Stocker and Hafen, 2000); and hyperplastic with constant cell size, such as Hippo, Salvador, Merlin, Warts (Pantalacci et al., 2003; Udan et al., 2003; Wu et al., 2003). At the same time, biochemical studies demonstrated that the gene products belong to two signal transduction pathways integrating environmental cues and regulating tissue growth: the mTOR and the Hippo kinase pathways (Ma et al., 2018; Liu and Sabatini, 2020). mTOR mainly senses nutrient availability, whereas the Hippo pathway mainly senses the physical environment. While TSC1, TSC2 and PTEN are negative regulators of the mTOR pathway, LOF mutants in positive regulators, such as Insulin Receptor Substrate (IRS), Phosphoinositide-3-Kinase (PI3K), Akt and mTOR itself, reduce both cell size and number (Leevers et al., 1996; Bohni et al., 1999; Verdu et al., 1999; Zhang et al., 2000; Gao et al., 2002; Zhang et al., 2003). Of note, adult flies lacking the *S6K* gene have the same cell number as wild type flies but their cell size is reduced, as measured in the eye and wing tissues (Montagne et al., 1999). The differential impact of growth mutants on cell size and cell number points to a loose relationship between these parameters. While the *Drosophila* fly genome contains a single *S6K* gene, two orthologous *RPS6KB1* and *RPS6KB2* genes exist in mammals, encoding the S6K1 and S6K2 proteins, respectively (Banerjee et al., 1990; Kozma et al., 1990; Gout et al., 1998; Shima et al., 1998; Lee-Fruman et al., 1999). Here, we will review the studies detailing the effectors of S6 kinases and the physiological alterations concomitant with the cell size control by this molecular program.

S6K and Akt belong to the AGC family of serine and threonine protein kinases (Pearce et al., 2010). They are activated by phosphorylation on their hydrophobic motifs and T-loop motifs by mTOR and 3-Phosphoinositide Dependent Protein Kinase (PDK1), respectively (Alessi et al., 1997; Alessi et al., 1998; Pullen et al., 1998; Hara et al., 2002; Kim et al., 2002; Sarbassov et al., 2005). However, mTOR resides in different protein complexes when it catalyzes S6K or Akt phosphorylation. While S6K is phosphorylated by mTOR complex 1 (mTORC1), Akt is phosphorylated by mTOR complex 2 (mTORC2). mTORC1 and mTORC2 differ in their sensitivity to the two main anabolic signals from nutrition: small nutrients molecules, such as glucose, amino acids and lipids which enter in circulation after food digestion, and growth factor peptides, such as insulin, that are secreted after food intake and coordinate nutrient usage in peripheral organs. mTORC1 activity requires signals from both nutrients and growth factors, while mTORC2 activity is relatively insensitive to nutrient levels (Liu and Sabatini, 2020). In addition, mTORC2 promotes mTORC1 activity, while being negatively regulated by mTORC1 in a retrocontrol mechanism. This cross-talk and differential sensitivity of mTORC1 and mTORC2 to the anabolic signals explain many physiological adaptations to nutrition. For instance, protein synthesis and cell growth are mainly upregulated by mTORC1 when both insulin and nutrients are present (Pham et al., 2000). Conversely, the metabolic action of insulin on nutrient uptake and gluconeogenesis is mainly regulated by mTORC2, with an excess of nutrients causing insulin resistance through the negative feedback loop of mTORC1 on mTORC2 (Fingar et al., 1993; Tremblay and Marette, 2001).

The convergence of nutrients and insulin signals for mTORC1 activation takes place at the lysosomal membrane, through the regulation of two small GTPase family members Ras Related GTP Binding A/B/C/D (RagA/B/C/D) and Ras Homolog Enriched In Brain (Rheb) that are important for mTORC1 localization and activity, respectively (Liu and Sabatini, 2020). It is becoming increasingly clear that the phosphorylation of protein substrates by mTORC1 is differentially sensitive to upstream regulators. Since the relative activity of mTORC1 towards S6K1 is low, this phosphorylation event is very sensitive to rapamycin treatment and nutritional perturbations that change mTORC1 activity through Rheb and Rag GTP loading (Chung et al., 1992; Kang et al., 2013). Conversely, the phosphorylation of other mTORC1 substrates, such as 4EBPs, is more constitutive and less sensitive to nutritional fluctuations as well as pharmacological inhibition by rapamycin (Choo et al., 2008). Moreover, phosphorylation of certain substrates, such as TFEB, is insensitive to the Rheb-dependent regulation of mTORC1 and exquisitely depends on the regulation at the level of RagC/D (Napolitano et al., 2020). It appears therefore that mTORC1 is capable to integrate a large variety of information from the environment and adapt the output towards distinct classes of substrates depending on the conditions.

S6 kinases are also extensively phosphorylated in the proline rich C-terminus region, a pseudosubstrate domain possibly exerting autoinhibitory effects on kinase activity (Mukhopadhyay et al., 1992; Dennis et al., 1998). The phosphorylation of the C-terminus region has been proposed to relieve the autoinhibitory regulation and expose the kinase for phosphorylation on the hydrophobic motifs and T-loop motifs required for activation and substrate docking. Several proline directed kinases can phosphorylate the C-terminus region of S6K1 *in vitro*, including Cyclin dependent kinases (Cdk) Cdk5 and Cdk1, Mitogen Activated protein kinases (MAPK) and mTOR itself (Mukhopadhyay et al., 1992; Arif et al., 2019). It is still unclear whether these kinases can compensate each other or whether they directly phosphorylate specific sites *in vivo*. This model of S6K1 regulation is consistent with the genetic epistasis experiments and pharmacological treatments by rapamycin derivatives, indicating that mTOR-mediated phosphorylation on the hydrophobic motif is absolutely required for S6K1 action on downstream substrates. However, a recent study proposes that Cdk5-mediated S6K1 phosphorylation on the C-terminus region may trigger a conformational switch that directs S6K1 kinase activity towards a different class of substrates (Arif et al., 2019). This appealing and original control still requires further work, namely the *in vivo* analysis of Cdk5 knockout mice and the generation of phospho-specific antibodies against the Cdk5-directed substrates. Of note, additional Cdk, such as Cdk4 and Cdk6, impinge on mTOR/S6K activity by affecting upstream regulators, such as the lysosomal complexes and TSC, rather than directly on kinase phosphorylation (Martinez-Carreres et al., 2019; Romero-Pozuelo et al., 2020).

Given the peculiar sensitivity of S6K1 to mTORC1 activity, it is not surprising that S6K1 deficient mice mimic a caloric restriction phenotype. They have low insulin levels in their blood, are hypersensitive to action of insulin on glucose tolerance, do not become obese after high fat diet and live longer (Pende et al., 2000; Um et al., 2004; Selman et al., 2009). Of note, all these physiological adaptations are accompanied by a reduction of cell size. The cell volume shrinkage is approximately 15%–35% depending on the cell type. This is less dramatic than the profound cell atrophy due to mTOR deletion, consistent with the idea that *S6K1* deletion does not induce autophagy. Similarly, *S6K* deletion in *Drosophila* flies rescues the overgrowth phenotype of the *Gigas-TSC2* mutation to the level of wild type tissues (Gao et al., 2002). The effect of S6K on cell size can be recapitulated *in vitro* after genome editing in cultured cells, pointing to a cell autonomous regulation of cell size by S6K1 (Bonucci et al., 2020). The decreased cell size is observed throughout different phases of cell cycle as well as in post-mitotic cells (Ohanna et al., 2005; Bonucci et al., 2020). *S6K1* deletion mimics the effect of rapamycin on cell size but not on cell proliferation (Ohanna et al., 2005; Dowling et al., 2010). Conversely, *4EBPs* deletion causes resistance to the anti-proliferative action of mTOR inhibitors without affecting cell size (Dowling et al., 2010).

**FIGURE 1:**
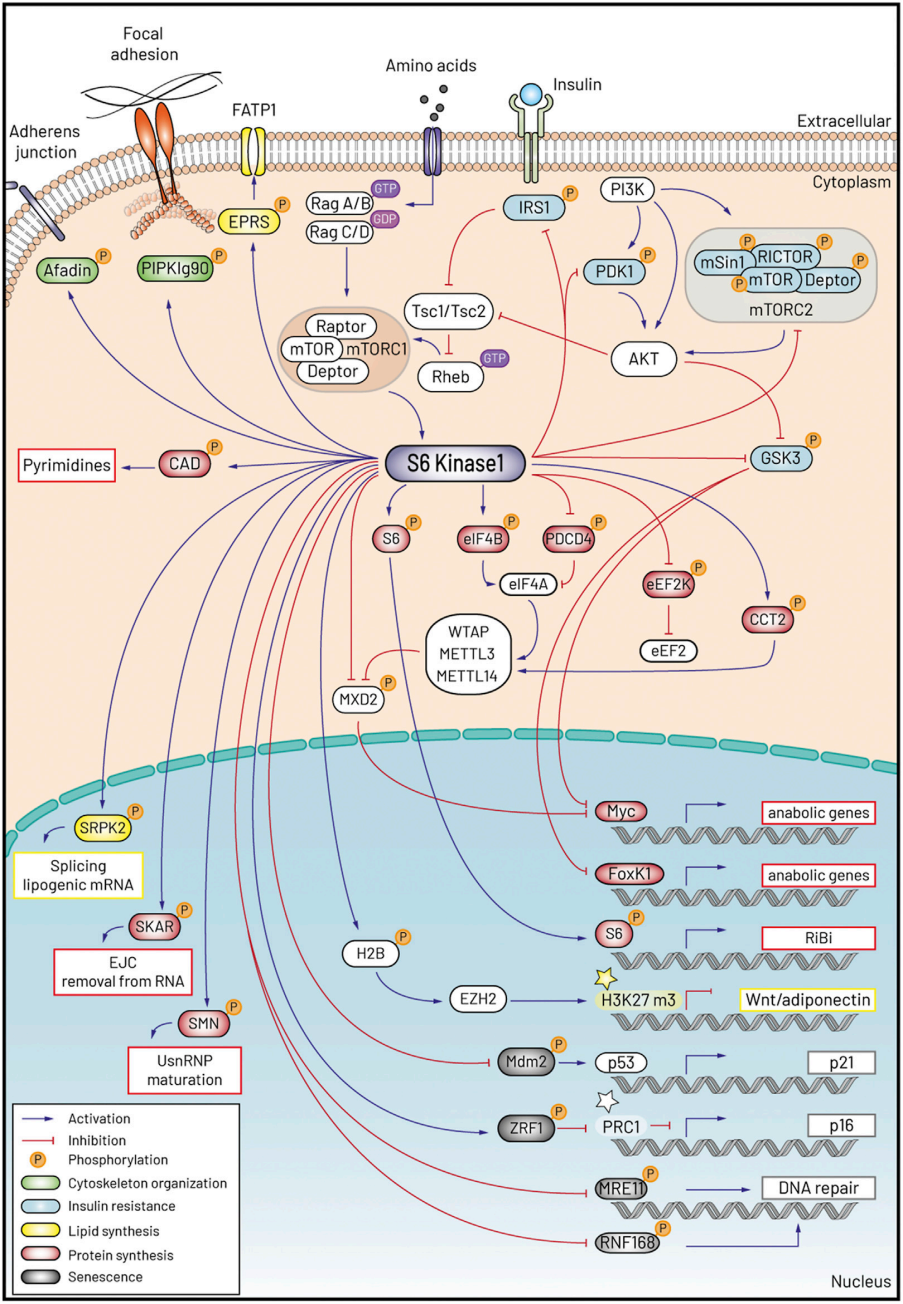
Schematic representation of S6K1 signaling and the five programs accounting for the control of cell size and ageing.

Cell size fluctuations have been recently proposed to directly affect senescence or stem cell potential (Neurohr et al., 2019; Lengefeld et al., 2021). We will now discuss how the S6K dependent molecular program may contribute to these coordinated responses: protein and lipid mass accumulation, insulin resistance, cytoskeletal dynamics and senescence. The elucidation of the S6K substrates is rapidly progressing thanks to the deep coverage of the phosphoproteomics analysis after rapamycin treatment, mTORC1 or S6K1 genetic deletion (Moritz et al., 2010; Hsu et al., 2011; Yu et al., 2011; Robitaille et al., 2013; Bonucci et al., 2020). A comprehensive and updated list of S6K substrates can be found at the PhosphoSitePlus online tool: https://www.phosphosite.org/substrateSearchViewAction.action?id=1012&type=Protein: the phosphorylation sites and consensus sequences are also adequately indicated there and are therefore omitted in this review. Figure 1 is a graphic representation of the S6K1 signaling network.

## Protein mass accumulation

### Protein synthesis

Substrates belonging to the protein synthesis machinery represent the most important class of S6K targets for both historic reasons and their high number. S6 kinases were initially isolated due to their enzymatic activity phosphorylating the ribosomes. Phosphorylation of the 40S ribosomal protein S6 (eS6 according to the novel nomenclature (Ban et al., 2014)) occurs in mammals and *Xenopus* at the C-terminus of the protein (Krieg et al., 1988; Wettenhall et al., 1992). *In vivo*, S6K1 and S6K2 are the main kinases responsible for eS6 phosphorylation, although residual eS6 phosphorylation by compensating p90 Ribosomal Protein S6 Kinase RPS6KA1-3 (RSK 1-3) downstream of Mitogen Activated protein kinases (MAPK, ERK1 and ERK2) can occur in an S6K null background (Pende et al., 2004). The evidence of eS6 phosphorylation playing a stimulatory role on protein synthesis is correlative and its function in mRNA translation remains elusive. Some insights on a physiological function of eS6 phosphorylation have arisen from the analysis of the *S6* ^−/−P^ knock-in mice where all the five phosphorylatable serines of eS6 are mutated to alanines (Ruvinsky et al., 2005). Although the phenotype of *S6* ^−/−P^ knock-in mice is milder than S6K1−/− mice, they also display small cell size of pancreatic beta cells and muscle cells, suggesting that eS6 phosphorylation participates in cell size control (Pende et al., 2000; Ohanna et al., 2005; Ruvinsky et al., 2005; Ruvinsky et al., 2009). If the phenotype of *S6* ^−/−P^ mice would be consistent with a stimulatory role of eS6 phosphorylation on protein synthesis, it is puzzling that *S6* ^−/−P^ cells from different tissues display higher protein synthesis rates than their wild type (WT) controls, arguing that eS6 phosphorylation has an inhibitory function on mRNA translation (Ruvinsky et al., 2005; Hsieh et al., 2010). Consistently, S6K1 deficient mice do not display a reduction in global protein synthesis (Mieulet et al., 2007). The mechanistic aspects of these effects have not been thoroughly investigated and it cannot be excluded that they arise from the activation of compensatory mechanisms. Some clues come from a recent study that by comparing the translation efficiency of WT and *S6* ^−/−P^ cells provides evidence that eS6 phosphorylation has a mild stimulatory effect on the translation of short mRNAs (Bohlen et al., 2021). Among those, however, an exception is that of 5TOP mRNAs that mainly encode ribosomal proteins and are translated more efficiently in *S6* ^−/−P^ than WT cells. In agreement with this observation, ribosomal protein levels, a proxy of ribosome content, are higher in *S6* ^−/−P^ as compared to WT cell (Bohlen et al., 2021), which is consistent with the difference in the rates of protein synthesis (Ruvinsky et al., 2005). An *in vivo* study employing *S6* ^P−/-^ mice indicates that in liver of animals fed after fasting eS6 phosphorylation mediates in part the stimulatory effects of S6K on the transcription of genes encoding RiBi (ribosome biogenesis) factors, implicated in the assembly and maturation of ribosomal subunits, contributing therefore to the increase in ribosome synthesis that ensues in response to feeding (Chauvin et al., 2014). It is likely that these effects are due to an extra-ribosomal function of eS6, although it cannot be excluded that phosphorylated ribosomes regulate the synthesis of factors that impact on Ribi mRNA levels.

The major hub of protein synthesis control by the mTORC1 pathway is the eIF4F (eukaryotic initiation factor 4F) complex that mediates during the initiation phase the recruitment to the mRNA of the 43S pre-initiation complex, i.e., the 40S ribosomal subunit bound to the ternary complex (eIF2-GTP-Met-tRNA), and the initiation factors eIF1, eIF1A, eIF3, and eIF5 (Merrick and Pavitt, 2018). eIF4F is composed by a scaffold, eIF4G, that interacts with the two other components of the complex, the cap binding protein eIF4E and the RNA helicase eIF4A. During initiation of protein synthesis, eIF4G interacts with the 43S preinitiation complex whereas the eIF4E moiety makes contact with the cap structure found at the 5′ end of mRNAs. This interaction is favoured by eIF4A that unwinds secondary structures proximal to the cap and facilitates therefore binding of the 43S complex to the mRNA. In this process the activity of eIF4A is greatly enhanced by the factor eIF4B (Merrick and Pavitt, 2018). Formation of the eIF4F complex is regulated in an mTORC1 dependent manner by growth factors and nutrients. Thus, under conditions of nutrient scarcity, members of the 4EBP (eIF4E binding protein) family efficiently compete with eIF4G for binding to eIF4E preventing therefore eIF4F complex formation and suppressing protein synthesis. Upon nutrient addition, phosphorylation of 4EBPs at several sites mainly by mTORC1, promotes their dissociation from eIF4E which becomes available for the formation of the eIF4F complex allowing an increase in the rates of protein synthesis (Merrick and Pavitt, 2018). A further layer of regulation on eIF4F formation is provided by S6K. This involves the phosphorylation and subsequent ubiquitin-dependent degradation of programmed cell death 4 (PDCD4), a protein that interferes with eIF4A activity and its binding to eIF4G (Dorrello et al., 2006). Furthermore, phosphorylation of eIF4B by S6K stimulates protein synthesis through a mechanism that may involve recruitment of eIF4B in proximity of eIF4F (Holz et al., 2005; Shahbazian et al., 2006).

Besides translation initiation, elongation is also subjected to regulation by the pathway. An inhibitory effect on protein synthesis occurs through phosphorylation and consequent inactivation of eukaryotic elongation factor 2 (eEF2) that, during elongation, catalyses the translocation of peptidyl-tRNA from the A to the P site of the translating ribosome (Dever and Green, 2012). The kinase responsible for eEF2 phosphorylation is the Ca2+/Calmodulin dependent kinase eEF2 kinase (eEF2K) (Kenney et al., 2014). This response is thought to promote cell survival under conditions of nutrient scarcity by limiting the highly energy-consuming process of protein synthesis (Leprivier et al., 2013; Faller et al., 2015). It has been shown that in response to mitogenic signals S6K phosphorylates and inactivates eEF2K, ensuring therefore high rates of protein synthesis (Wang et al., 2001; Kenney et al., 2014).

### RNA metabolism

S6K might regulate specific mRNA translation through the regulation of their metabolism, including mRNA methylation and splicing. It has been recently shown that in *TSC2*−/− cells, S6K through the eIF4A-eIF4B axis promotes the translation of the mRNA encoding Wilms tumor 1-associated protein (WTAP), the scaffold component of the m^6^A methyltransferase complex (MTC). This in turn results in formation of m^6^A by the MTC components m^6^A methyltransferase like proteins 3 and 14 (METTL3 and METTL14) on, among others, the mRNA encoding the c-myc suppressor MAX dimerization protein 2 (MXD2), triggering its degradation and leading to activation of c-myc transcriptional activity (Cho et al., 2021). MXD2 can also be directly phosphorylated by S6K1, an event promoting protein ubiquitination and degradation (Huang et al., 2018). A role of eIF4B phosphorylation by S6K in directly promoting translation of c-myc mRNA has also been documented in *TSC2*−/− cells (Csibi et al., 2014). Taken together, these findings converge on the Myc transcriptional program in mediating some of the growth-promoting actions of S6K.

Moreover, S6K intervenes in the final maturation steps of mRNAs. As introns are removed from pre-RNAs during splicing, a complex forms twenty four nucleotides upstream of exon-exon junctions known as EJC (Exon-Junction complex). mRNAs associate then to the nuclear CAP binding complex (CBP) and are exported from the nucleus into the cytosol. Here mRNAs, still bound to CBP, are inspected by the so-called pioneer round of translation, a quality control step during which EJC are removed unless an upstream premature translation stop codon is present. Successful removal of the EJC commits the mRNA toward productive protein synthesis (Woodward et al., 2017). It has been shown that S6K1 Aly/REF-like target (SKAR), an RNA binding protein phosphorylated by S6K1 (Richardson et al., 2004), is a component of the EJC (Ma et al., 2008). In response to Insulin, S6K1, by interacting with SKAR, is recruited to CBP-bound mRNPs and presumably stimulates the inspection of the associated mRNAs by the pioneer round of translation providing transcripts to support protein synthesis (Ma et al., 2008). It is interesting that knock down of SKAR results in a small cell size phenotype (Richardson et al., 2004). It will be important to analyse the role that SKAR phosphorylation by S6K plays on regulation of cell size as well as on SKAR function as a component of the EJC. Additional splicing factors controlled by S6K will be discussed in the next chapter.

### Protein folding

Growth control by mTOR and S6K also relies on protein folding. In *Drosophila*, the overgrowth phenotype of TOR activation is suppressed by depletion of subunits of chaperonin containing tailless complex polypeptide 1 (CCT), a complex that assists the folding of about 10% of newly synthesized cytosolic proteins (Kim and Choi, 2019). TOR promotes transcription of CCT genes. Of note, CCT subunits are important to maintain the levels of METTL3 and METTL14 in MTC, which contribute to the inhibition of autophagy by catalysing formation of destabilising m6A on mRNAs encoding autophagy effectors (Tang et al., 2021). Some aspects of this regulatory circuit are conserved in mammalian cells. A possible role of S6K in regulating both CCT and MTC functions is supported by the finding that the CCT2 subunit is phosphorylated by S6K (Abe et al., 2009). Interestingly a CCT2 phosphomutant fails to rescue the proliferation defect caused by depletion of CCT2 in human mammary epithelial cells. S6K also phosphorylates ZRF1, the HSP40 component of the ribosome-associated complex (RAC), a universally conserved broad specificity chaperone system that interacts with the 60S ribosomal subunit assist co-translational folding of nascent polypeptides as they emerge from the tunnel of the ribosome (Barilari et al., 2017). Furthermore, studies in yeast suggest that ZRF1 has a role in maintaining protein synthesis fidelity by interacting with the 40S subunit at a region that is involved in decoding (Lee et al., 2016). It will be important to determine the functional relevance of this phosphorylation event on protein synthesis fidelity.

### Nucleotide biosynthesis

A major cellular function involved in cell growth is the synthesis of precursors employed in macromolecular synthesis. These include the pyrimidine and purine bases that are then employed for the synthesis of nucleosides, the components of DNA and RNA (Zhu and Thompson, 2019). Recent work from the Manning laboratory has shown that *de novo* synthesis of both pyrimidines and purines is driven by the mTOR pathway (Ben-Sahra et al., 2013; Ben-Sahra et al., 2016). Downstream of mTOR, S6K plays a crucial role on pyrimidines synthesis through the stimulatory phosphorylation of carbamoyl-phosphate synthetase 2, aspartate transcarbamoylase, dihydroorotase (CAD), the enzyme that catalyses the first three steps of *de novo* pyrimidine synthesis (Ben-Sahra et al., 2013; Robitaille et al., 2013).

## Lipid mass accumulation

Interestingly, phosphorylation of splicing and translational machineries has been implicated in one of the most striking cellular responses to S6K1 activity, the fat accumulation. In *Drosophila*, a lipogenic program is sufficient to alter cell size (Porstmann et al., 2008). Although S6K1 has been proposed to directly phosphorylate and regulate the expression of the master regulator of lipid gene expression, the Sterol Regulatory Element-Binding Protein 1 (SREBP1), the experimental evidence is not conclusive (Lewis et al., 2011; Yecies et al., 2011). Two recent studies suggest alternative SREBP1- and transcription-independent mechanisms for the regulation of fat metabolism, involving the Serine-arginine rich protein kinase 2 (SRPK2) and the glutamyl prolyl tRNA synthetase EPRS. SRPK2 regulates serine arginine rich proteins (SR), whose function is to catalyze mRNA splicing by binding exons and recruiting small nuclear ribonucleoproteins (snRNPs) (Lee et al., 2017). S6K1 dependent phosphorylation of SRPK2 in cooperation with an additional Casein Kinase 1 (CK1)-dependent site upregulates nuclear localization of the protein. Nuclear SRPK2 promotes efficient intron splicing of acly, acss2, hmgcs1, mvd, fdft1, scd1, fasn mRNA, thus increasing the stability and the levels of these mRNA coding for proteins involved in lipid synthesis. Conversely, the S6K1-dependent phosphorylation of EPRS acts as a switch for the function of the protein, that becomes a partner of Long-Chain Fatty Acid Transport Protein (FATP1), promoting long chain FA uptake and TG synthesis (Arif et al., 2017). Similar to S6K1 deficient mice, EPRS phosphomutant mice also have reduced fat mass and increased longevity, indicating a participation of the post-translational modification in these phenotypes (Arif et al., 2017). Moreover, when S6K1 receives activating input by Cdk5, additional proteins involved in lipid synthesis can be phosphorylated, including cortactin, lipocalin 2 and coA synthase (Arif et al., 2019). Another original mechanism for the control of lipid synthesis is through epigenetic modifications triggered by S6K1-dependent Histone 2B (H2B) phosphorylation (Yi et al., 2016). In adipocytes, S6K1 can translocate to the nucleus upon treatment with the adipogenic factor Bone Morphogenetic Protein 4 (BMP4). The H2B phosphorylation recruits the Histone-Lysine N-Methyltransferase EZH2 and favours H3K27 trimethylation (H3K27m3), thus suppressing Wnt ligand expression which acts as a brake for adipogenic commitment. The expression of fat-regulating hormones in adipocytes and pancreas, such as adiponectin, insulin and glucagon, may also undergo a similar epigenetic regulation (Yi et al., 2018; Yi et al., 2022).

## Insulin sensitivity

S6K1 is involved at multiple levels in the above-mentioned negative feedback mechanism by which an excess of nutrients through the mTORC1/S6K1 axis dowregulates insulin signaling and mTORC2 activity. First, S6K1 acts at the level of IRS proteins which initiate insulin signaling. IRS phosphorylation on Ser and Thr residues by S6K1 and additional kinases contributes to shut down the signal by promoting protein degradation or decreasing the interaction with the receptors (Harrington et al., 2004; Tremblay et al., 2007). Consistently, the increase in insulin sensitivity of S6K1-deficient mice correlates with a decrease in IRS1 Ser/Thr phosphorylation (Um et al., 2004). Second, S6K1 phosphorylates multiple proteins in mTORC1 and mTORC2. S6Ks phosphorylate mTOR itself, however the functional relevance of these phosphorylation sites is unclear (Holz and Blenis, 2005). S6Ks also phosphorylate two components of the mTOR complex 2 (mTORC2), namely rapamycin-insensitive companion of mTOR (RICTOR) and stress-activated MAPK-interacting protein 1 (SIN1) (Dibble et al., 2009; Julien et al., 2010; Treins et al., 2010; Liu et al., 2013), and the Deptor protein present in both mTORC1 and mTORC2 (Gao et al., 2011; Zhao et al., 2011). Although there are some controversies on the function of each phosphorylation event, there is a general consensus that S6Ks act by inhibiting the mTORC2 substrate Akt. Phosphomimetic SIN1 mutants are unable to interact with RICTOR and mTOR, while phospho-ablative mutants increase Akt phosphorylation (Liu et al., 2013). Similarly, two studies have shown a mild inhibitory effect of RICTOR phosphorylation on AKT activity (Dibble et al., 2009; Julien et al., 2010). The reduced half life of Deptor upon its phosphorylation may also concur to decreased Akt activation (Gao et al., 2011; Zhao et al., 2011).

Another level by which S6K1 may suppress Akt activity is through the phosphorylation of PDK1 on the pleckstrin homology domain that impairs the interaction between PDK1 and Akt (Jiang et al., 2022). Interestingly, if S6K1 has clearly a negative role on the metabolic action of insulin and Akt, it can compensate the Akt action on some substrates that are important growth regulators, such as Glycogen Synthase Kinase 3 (GSK3). S6K1 can phosphorylate GSK3 in conditions of mTORC1 overactivation (Zhang et al., 2006), and thus relieve the GSK3-dependent inhibition of two transcription factors promoting growth, c-Myc and Forkhead Box Protein K1 (FoxK1) (He et al., 2018).

## Senescence

Rapamycin treatment has a striking efficacy in delaying the senescence program in cultured cells and preserving their proliferative potential (Leontieva and Blagosklonny, 2013, 2014; Leontieva et al., 2013, 2014; Neurohr et al., 2019; Lengefeld et al., 2021). Primary cells undergoing senescent insults of DNA damage, replication stress, oncogenic mutations and constitutive mTOR activation are protected by rapamycin. Pharmacological inhibition of S6K1 or genetic knock-down/knock-out also rescues the senescent program (Leontieva et al., 2013; Barilari et al., 2017). In TSC1 deficient fibroblasts, S6K1 regulates the expression of the cell cycle inhibitor p16 (Barilari et al., 2017). This is accompanied by the S6K1-dependent phosphorylation of Zrf1, a protein containing a SANT domain for DNA and histone binding and interacting with inhibitor of differentiation 1 (Id1) (Shoji et al., 1995; Richly et al., 2010). Zrf1 has been shown to antagonize the action of polycomb repressive complex 1 (PRC1) that ubiquitinates H2A and silences the *Ink4a/ARF* locus encoding p16 (Richly et al., 2010). In addition, the Zrf1 partners Id proteins can also suppress the activity of ETS1 and ETS2 transcription factors, which are potent activators of p*16* transcription (Shoji et al., 1995). As outlined above, Zrf1 has also cytosolic functions as a co-chaperon forming the ribosome-associated complex (RAC). Although the phosphomutant of Zrf1 delays the senescence program (Barilari et al., 2017), how this post-translational modification affects the molecular function of Zrf1 has not been addressed yet.

S6K1 activity also modulates the DNA damage response, relevant to the induction of senescence. In cells exposed to DNA damaging agents and nutrient-rich conditions, S6K1 contributes to the phosphorylation and cytosolic retention of Mouse double minute homolog 2 (MDM2), the E3 ubiquitin ligase controlling the degradation of p53 (Lai et al., 2010). This potentiates the p53-dependent transcriptional response to DNA damaging agents, and in particular may explain how nutrients and growth factors can favour the pro-apoptotic and pro-senescent function of p53. Moreover, the S6K1-dependent phosphorylation of meiotic recombination 11 protein (MRE11) and Ring Finger Protein 168 (RNF168) triggers the degradation of these proteins crucial for DNA end resection and homologous recombination in the repair of double strand breaks (Piscitello et al., 2018; Xie et al., 2018). This impairs the DNA damage checkpoint and favours the entry of primary fibroblasts into senescence.

## Cytoskeleton dynamics

The S6K1-dependent hypertrophy of *TSC1* mutant kidney epithelial cells correlates with misorientation of cell division leading to cyst deformation of renal tubules (Bonucci et al., 2020). However, micropattern experiments to measure the angle of cell division in single cells demonstrate that aberrant cell size is not sufficient to alter the mitotic angle. The defect of oriented cell division can be observed when cells are adjacent to each other in an epithelial layer. This suggests the importance of adhesion cues in the correct determination of planar polarity. Of note, a comprehensive phosphoproteomics analysis of the inner medullary collecting duct cell line mIMCD3 edited in the *TSC1* and *S6K1* genes revealed a broad list of putative S6K1 substrates involved in the regulation of the actin cortex and cell adhesion. These include slingshot protein phosphatase 1 (SSH1), phosphatase for the actin depolymerase cofilin; microtubule actin crosslinking factor 1 (Macf1), spectraplakin binding actin and microtubules; phosphatidylinositol 4-Kinase α (PI4K), regulating endocytic trafficking; myosin phosphatase-targeting subunit 1 and 2 (MyPT1 and MyPT2), myosin phosphatases regulating actomyosin contractility; missing in metastasis protein (Mtss1), cortactin-interacting protein. Among the phosphopeptides, Afadin, an actin binding and adapter protein regulating the nectin and cadherin adhesion systems, has been validated as a novel S6K1 substrate. Afadin phosphorylation alters Adherens Junctions in kidney epithelial cells and favours cell migration of breast cancer cells (Elloul et al., 2014; Gao et al., 2017). Of note, it has been suggested that Afadin phosphorylation may also be involved in the negative feed-back regulation of insulin action in adipocytes (Lundh et al., 2019), and Afadin phospho-mutant mice display a mild amelioration of glucose homeostasis at the early stages of high fat diet (Tozzi et al., 2022). Consistent with an important role of the mTOR/S6K1 pathway on cell adhesion, S6K1 phosphorylation down-regulates PI 4 phosphate 5 kinase type I g (PIPKIg90), which mediates together with talin the assembly of focal adhesion (Jafari et al., 2016). As a result of S6K1 action on PIPKIg90, cells become more prone to matrix degradation and invasion.

## Future perspectives on the spatial control of cell growth and ageing responses

It has been recently proposed that cell size alterations may influence important cell fate decisions, such as senescence or regenerative responses (Neurohr et al., 2019; Lengefeld et al., 2021). It is likely that mTOR and S6K affects physical properties of the cells and define spatially organized microdomains, therefore affecting molecular machineries involved in growth and ageing. Several evidences are consistent with this possibility, though the causal relationships will require further studies. It has been shown that inhibition of mTOR by rapamycin treatment is associated with a decrease of macromolecular crowding and viscosity of the cytosol due to loss of ribosomes, a result of suppression of ribosome biogenesis and increased ribophagy (Delarue et al., 2018). Although it is not defined whether the decrease in ribosome contents is responsible of the decrease in cell size caused by rapamycin, it is clear that the ensuing dilution of the cytosol increases the diffusion of macromolecules. This may favour signaling processes but also impair the assembly of biomolecular condensates. The disproportionate growth of senescent cells, which is associated with a gradual loss of biosynthetic capacity, may also lead eventually to the dilution of the cytosol (Neurohr et al., 2019). The implication of S6K in this phenomenon is suggested by the observation that S6K inactivation prevents senescence of *TSC1*−/− cells (Barilari et al., 2017). S6K might contribute to a level of cytoplasmic crowding appropriate to regulate the on-demand formation of biomolecular condensates that perform crucial cellular functions. This would require a correct balance between the synthesis of crowding agents and other S6K dependent and independent functions that contribute to cell growth. Loss of this coupling would be expected to lead then to variations in the concentration of crowder and consequent alterations in cellular homeostasis.

In recent years, S6K activity has been implicated in the formation of biomolecular condensates, defined as dynamic membraneless subcellular compartments with liquid like properties which perform specific functions and assemble through phase separation. These include among others, nucleoli, Cajal bodies, promyelocytic leukaemia bodies (PML) in the nucleus, and P bodies, stress granules, or synaptic densities in the cytosol (Banani et al., 2017). For instance, stress granules (SGs) formation in response to oxidative stress and heath shock is under the control of the mTOR pathway and sensitive to S6K inhibition (Sfakianos et al., 2018). Although the mechanism has not been elucidated, it probably implicates phosphorylation of SG components that modulate protein-protein interactions and favour the formation of the condensates. Another example is the phosphorylation of the Survival motor Neuron protein (SMN) by S6K. This promotes efficient condensation by liquid liquid phase separation of the SMN complex in Cajal bodies (CBs) (Schilling et al., 2021), thus favouring the final maturation of ribonucleoproteins containing small nuclear U RNAs (UsnRNPs) and assisting the different steps of pre-mRNA splicing (Raimer et al., 2017). Interestingly SMN has SMN-complex independent functions. It has been shown that in fibroblasts a population of SMN is found associated with caveolin at the plasma membrane (Gabanella et al., 2016). Under conditions of recovery from energy stress, SMN at newly-formed membrane protrusion mobilizes inactive membrane-bound ribosomes that are then engaged in local protein synthesis in order to provide proteins required for the stabilization and maintenance of these structures. It will be interesting to determine the dependency of this process on the mTOR/S6K pathway, as well as the elucidation of additional microdomains influenced by these kinases.

As outlined in the previous chapter, the control of cytoskeleton dynamics may present an underappreciated mechanism pervading the whole cell and coordinating biological responses in relationship with cell volume regulation by S6K. In addition, specific metabolite levels and pathway activities may underlie cell volume alterations. In skeletal muscle, it is well established that muscle fiber types relying on different energy sources have different sizes (van Wessel et al., 2010; Bourdeau Julien et al., 2018). Mitochondrial functionality has been associated with the establishment of an optimal cell size (Miettinen et al., 2014; Miettinen and Bjorklund, 2017). Of note, the small size of the *S6K1* mutant muscles correlate with alterations in the AMP/ATP ratios (Aguilar et al., 2007), and AMPK activity is a direct target of S6K (Dagon et al., 2012). In addition, the phosphorylation of Bcl-2-associated agonist of cell death (BAD) by Akt and S6K might be involved in reprogramming energy metabolism (Harada et al., 2001; Gimenez-Cassina et al., 2014). Future studies will address the following outstanding questions: What physical properties of the cell, metabolic status and cystoskeleton modifications may contribute to the whole cell sensing of its size in relation with S6K activity? What S6K1 specific substrates, that cannot be compensated by S6K2 or Rsk activities, dictate the cell size adaptations? Can these mechanisms be targeted in age-related pathological conditions?
